# Organized Pneumonia Secondary to Increasing Doses of Temozolomide

**DOI:** 10.7759/cureus.318

**Published:** 2015-09-09

**Authors:** Pedro J Marcos, Angélica Consuegra Vanegas, María Matachana Martínez, Lourdes Cordero Lorenzana, Iria Vidal García, Carmen Montero Martínez

**Affiliations:** 1 Servicio de Neumología. Instituto de Investigación Biomédica de A Coruña (INIBIC), Complejo Hospitalario Universitario de A Coruña (CHUAC), Sergas. Universidade da Coruña (UDC).; 2 Servicio de Neumología. Instituto de investigación Biomédica de A Coruña (INIBIC), Complejo Hospitalario Universitario de A Coruña (CHUAC), Sergas. Universidade da Coruña (UDC).; 3 Unidad de Cuidados Intensivos. Instituto de investigación Biomédica de A Coruña (INIBIC), Complejo Hospitalario Universitario de A Coruña (CHUAC), Sergas. Universidade da Coruña (UDC).

**Keywords:** glioma, organized pneumonia, temozolomide, side effects

## Abstract

Surgery, radiotherapy (RT), and chemotherapy have a role in the control of tumor growth, progression, and recurrence in high-grade gliomas. Temozolomide has been incorporated as the main chemotherapy agent for managing these tumors. Here, we present a case of a patient who developed a severe organizing pneumonia after increasing doses of temozolomide for a high-grade glioma.

## Introduction

Low-grade gliomas have a long natural history, which often ends with the transformation to high-grade gliomas. Surgery, radiotherapy (RT), and chemotherapy have a role in tumor growth control, recurrence, and progression to high-grade tumors [1]. Temozolomide, an alkylating agent that is administered orally, has been incorporated as the main chemotherapy agent for managing these tumors. We present a case of a patient who developed a severe organizing pneumonia after increasing doses of temozolomide and a literature review of this uncommon adverse effect.

## Case presentation

A 50-year-old male was admitted in February 2014 to our hospital with fever, chills, shortness of breath, and weakness. The symptoms had begun two months earlier and worsened in a few days. Thirteen years before, he had been treated for a low-grade cerebral oligodendroglioma with complete resection and radiation therapy (RT). Ten years later, the patient was diagnosed with a tumor relapse and received treatment with surgery, RT, and a chemotherapy regimen based in temozolomide (100 mg a day) and bevacizumab (600 mg once every two  weeks). He continued with this treatment until October 2014 when the bevacizumab was withdrawn. The patient continued with the temozolomide monotherapy, increasing its dose from 100 mg a day to 240 mg a day. Signed informed patient consent was obtained.

**Figure 1:**
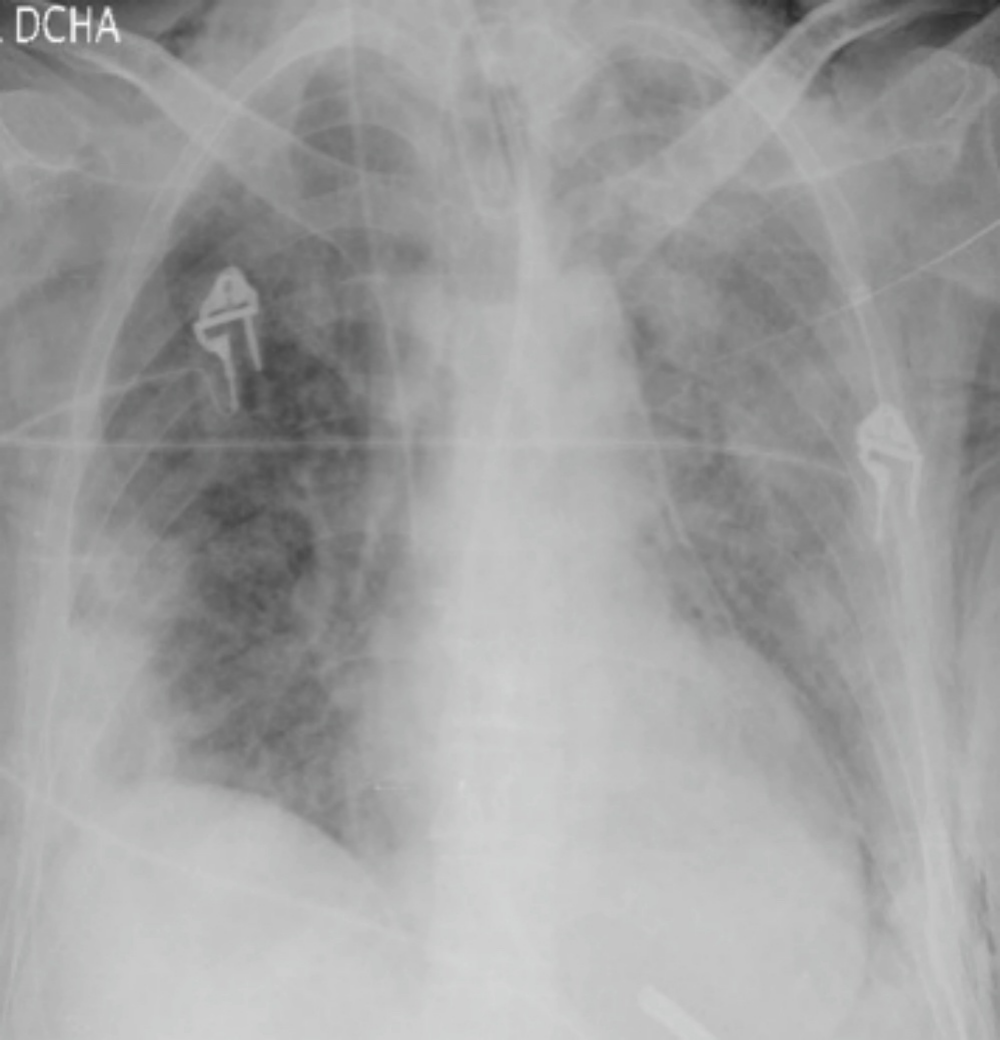
Chest x-ray at hospital admission Chest x-ray showing bilateral pulmonary infiltrates

**Figure 2:**
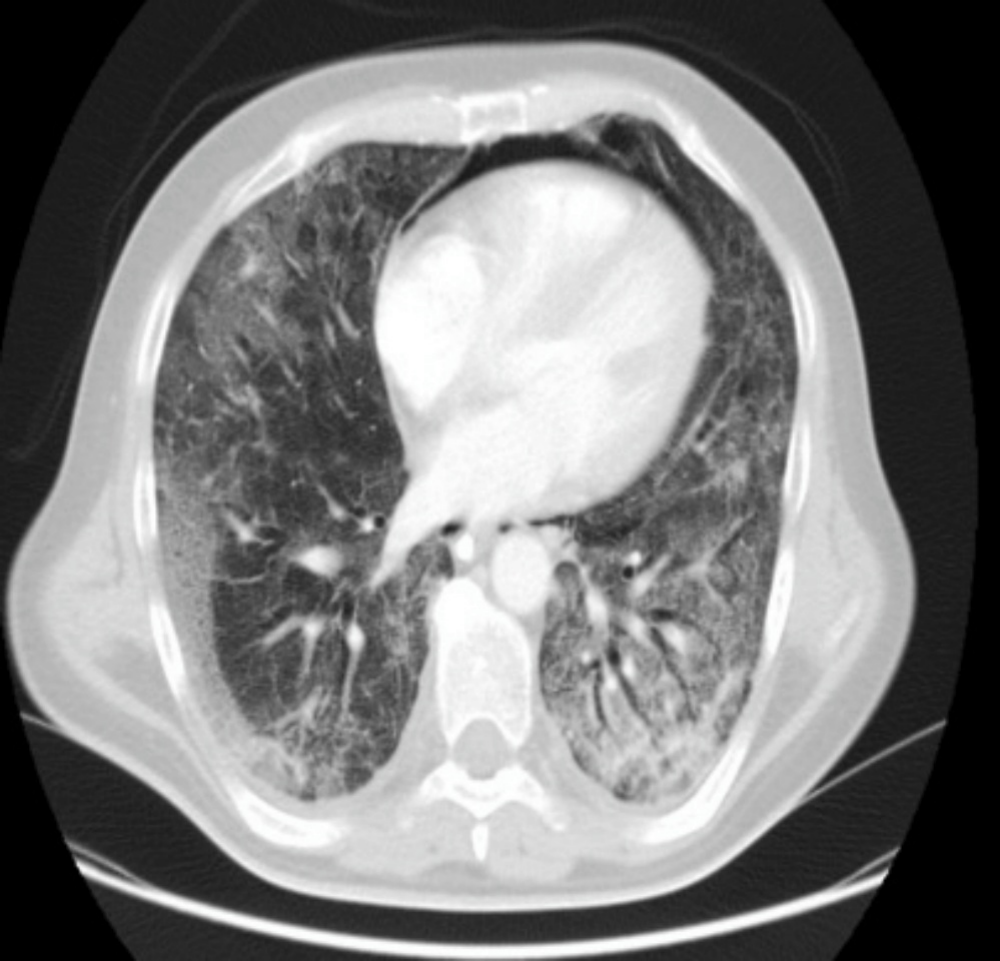
CT scan at hospital admission CT scan showing bilateral ground glass opacities and crazy-paving pattern

**Figure 3:**
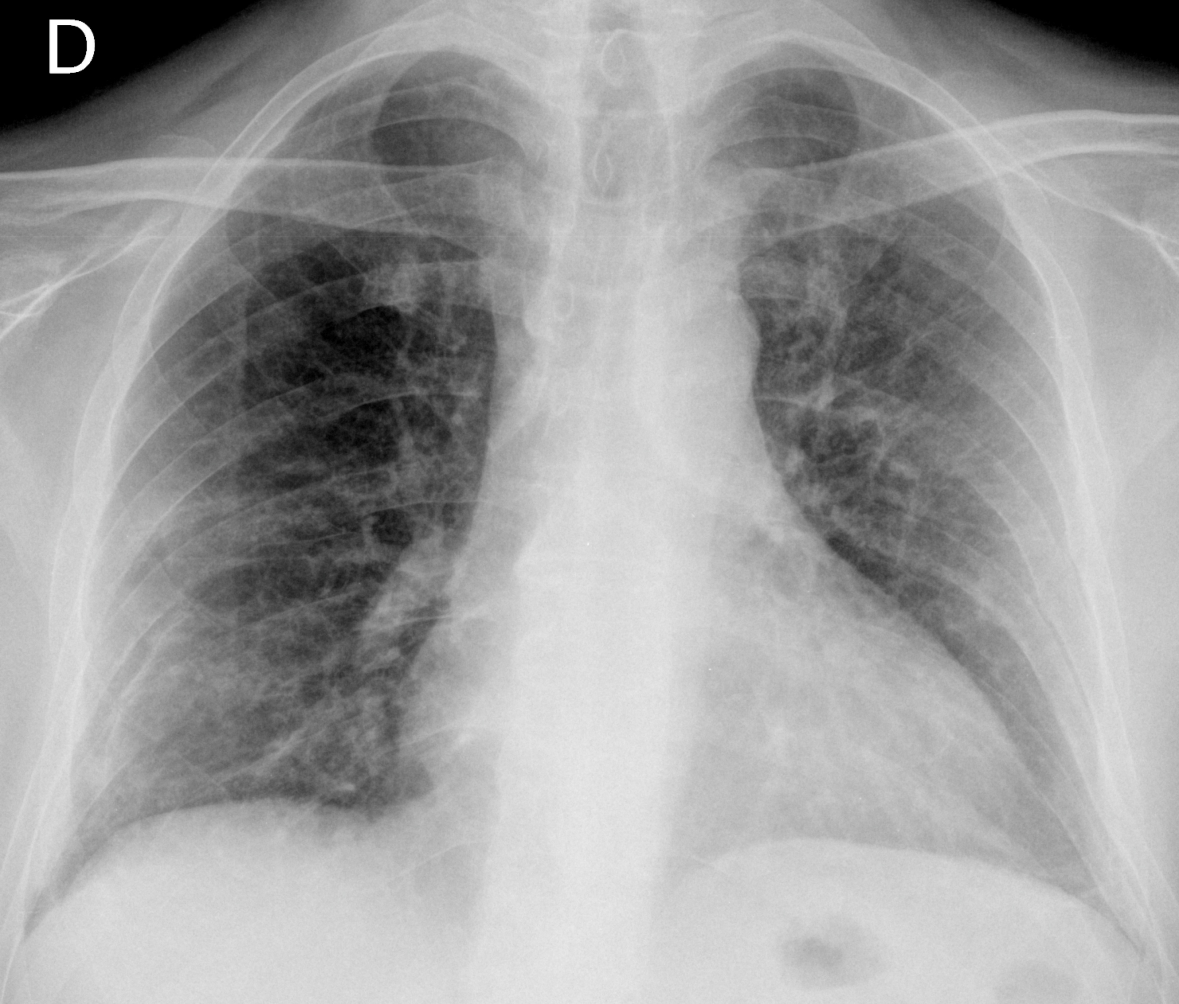
Chest x-ray after one month of steroid treatment

On physical examination, he was hemodynamically stable with a respiratory frequency of 27 breaths per minute (bpm) and bilateral crackles on lung auscultation. Neurologic exploration was normal. Arterial blood gases while breathing room air showed an hypoxemic respiratory failure and chest radiography showed bilateral parenchymal infiltrates (Figure 1). With the clinical suspicion of community-acquired pneumonia, an antibiotic regimen based on ceftriaxone and levofloxacin was initiated. On day two, the patient's clinical status worsened with tachycardia at 112 beats per minute (bpm), tachypnea at 40 bpm, and progression of the respiratory failure with an oxygen saturation of 77% with a fraction of oxygen inspired (FiO2) of 0.5. The patient was admitted to the intensive care unit (ICU). In the ICU, the patient needed invasive mechanical ventilation. A broad-spectrum empirical therapy was initiated with imipenem, amikacin, linezolid, trimethoprim-sulfamethoxazole, and oseltamivir for suspected septic shock in an immunosuppressed patient. A thoracic computed tomography (CT) scan was done, which showed multiple ground glass opacities with patchy involvement of all lung fields and thickening of the interlobar and interlobular septum, confirming a crazy-paving pattern (Figure 2). A fiberoptic bronchoscopy with bronchoalveolar lavage (BAL) was done; it revealed a cytological analysis with 35% lymphocytes, 25% neutrophils, and 3% eosinophils. Otherwise, BAL cytology showed no malignant cells or opportunistic pathogens with the usual staining done by our lab. Blood, urine, tracheal aspirate culture for bacteria, fungi, polymerase chain reaction for respiratory viruses, and *Pneumocystis jirovecii* were also negative. Due to the poor response to antibiotic therapy, the negative microbiological tests, the persistence of the radiological pattern, the respiratory failure despite treatment with the BAL findings, the history of treatment with temozolomide, an already-described drug with pulmonary effects, and the recent increase in the dose over the past weeks before the actual episode, the diagnosis of organized pneumonia was established. Methylprednisone was initiated at a dose of 500 mg/day for three days and then at a dose of 1 mg/kg/day. Forty-eight hours later, the patient had an important clinical recovery with significant improvement in respiratory symptoms. A radiological control was made one month later and it showed an almost complete resolution of the pulmonary infiltrates (Figure 3).

## Discussion

Temozolomide is a second generation-alkylating agent, which in recent years has been used mainly in the chemotherapy of brain tumors. Organized pneumonia (ON) is a condition characterized by subacute symptoms of fever, dyspnea, cough, and myalgia, as well as the presence of patchy consolidation and ground glass image in a CT scan. It may be idiopathic or secondary to connective tissue diseases, infections, drugs, or radiotherapy. The diagnosis is based on clinical and radiological suspicion and histologic confirmation by transbronchial or surgical biopsy. Fortunately, however, it has been described that a key feature of organized pneumonia is its excellent response to treatment with high doses of corticosteroids and complete resolution in weeks as described in the case presented above [2-3].

Two Phase 2 studies reported pneumonitis as a complication in 2-5% of subjects who received temozolomide [4-5]. In 2007, Maldonado, et al. published the first case of organized pneumonia secondary temozolomide. After establishing it as a diagnosis of exclusion, they started treatment with high doses of steroids, and the drug was discontinued four weeks later when the patient experienced complete relief of respiratory symptoms and radiological resolution [6]. To date, there have been a total of five cases described, which are summarized in Table 1.

**Table 1:** Temozolomide-associated pneumonitis cases MTP: metilprednisolone. TBB: transbronchial biopsy. GBM: glioblastoma multiforme.

Author	Age	Tumor	Temozolomide dosage	Treatment duration	TBB	Steroid dosage	Evolution
Maldonado [6] ; 2007	88	GBM	200 mg/day	6 months	Yes	Prednisone 1 mg/Kg/day	Recovery
Guillaminault [7]; 2008	56	GBM	225 mg/day	4-5 months	No	Prednisone 60 mg/day	Recovery
Koschel [8]; 2009	54	GBM	75 md/day	2 months	Yes	Prednisone 40 mg/day	Recovery
Kim [9]; 2012	56	GBM	200 mg/day	4 months	Yes	MTP 500 mg/day 5 days and MTP 1 mg/Kg/day	Recovery
Balzarini [10]; 2014	67	GBM	225 mg/day	3 months	Yes	X	Dead

Although in our case we did not have the histologic confirmation, the clinical-radiological presentation and the excellent response to high-dose corticosteroids following an increased dose of temozolomide, a potential pneumotoxic agent, we assumed the diagnosis to be organized pneumonia. Even when the patient was taking temozolomide for some years, we hypothesized that organizing pneumonia was related with the increasing dose of the drug, which could have led to the pneumotoxicity.

## Conclusions

Given the increase of therapies like temozolomide today, clinicians should consider organized pneumonia in the differential diagnosis, so that once infectious complications have been ruled out, an early corticosteroid treatment should be initiated in order to achieve the recovery in these patients.
